# Preduodenal Portal Vein Associated With Intestinal Malrotation and Jejunal Atresia

**DOI:** 10.7759/cureus.16467

**Published:** 2021-07-18

**Authors:** David J Zula, Adelene Y Houlton, Ramesh M Nataraja, Maurizio Pacilli

**Affiliations:** 1 Department of Paediatric Surgery, Monash Children's Hospital, Melbourne, AUS

**Keywords:** preduodenal portal vein, jejuna atresia, malrotation, duodenal obstruction, neonatal bowel obstruction

## Abstract

Preduodenal portal vein (PDPV) is a rare congenital anomaly commonly associated with other gastrointestinal abnormalities. We report the case of a female neonate with a PDPV, intestinal malrotation and jejunal atresia. This is the second account of this association reported in the literature. The previously reported case underwent a gastroduodenostomy to correct the presumed duodenal obstruction caused by the PDPV. In our case, the PDPV was not corrected and the child remains well and asymptomatic. We propose that in this rare association, the PDPV is not a cause of obstruction and does not need correction.

## Introduction

Preduodenal portal vein (PDPV) is a rare congenital anomaly, first described by Knight in 1921 [1]. The portal vein develops from the vitelline venous system and, in the foetus, loops around the developing duodenum with an anterior (left) and posterior (right) limb. In normal development, the anterior limb involutes, leaving the posterior limb to form the portal vein. However, if the posterior limb involutes leaving the anterior limb to form the portal vein, a PDPV results [1,2]. PDPV is a rare cause of duodenal obstruction [3,4] and is associated with intestinal malrotation; in one study, 64% of 323 patients with PDPV were associated with intestinal malrotation [5]. However, there is only one case-report in the literature of a neonate with the association of PDPV, intestinal malrotation and jejunal atresia [6]. In this previously reported case, the neonate underwent correction of the jejunal atresia and at a second operation had a Ladd’s procedure and a gastroduodenostomy.

Here we report the second case of a neonate with a PDPV, intestinal malrotation and jejunal atresia where the PDPV was not corrected.

## Case presentation

**Figure 1:**
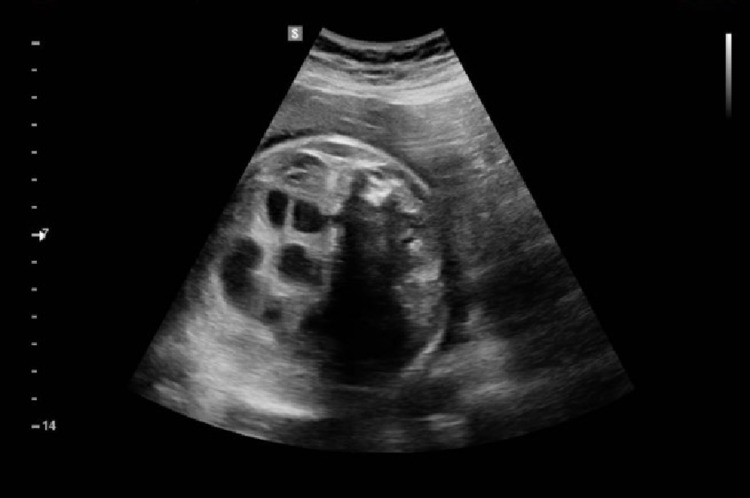
Antenatal ultrasound at 31/40 gestation showing normal calibre of distal bowel with dilated proximal bowel loops

On day 1 of life, shortly after an uneventful vaginal delivery at 36+6 weeks of gestation, a female neonate was referred to the surgical team. The parents had received antenatal counselling by the paediatric surgical team at 31 weeks of gestation for distended proximal bowel loops and a likely diagnosis of intestinal atresia (Figure 1). No other anomalies were identified on antenatal ultrasounds. The infant had a moderately distended abdomen and bilious fluid aspirated from the nasogastric tube. A plain abdominal radiograph showed multiple dilated small bowel loops with absent distal bowel gas.

**Figure 2:**
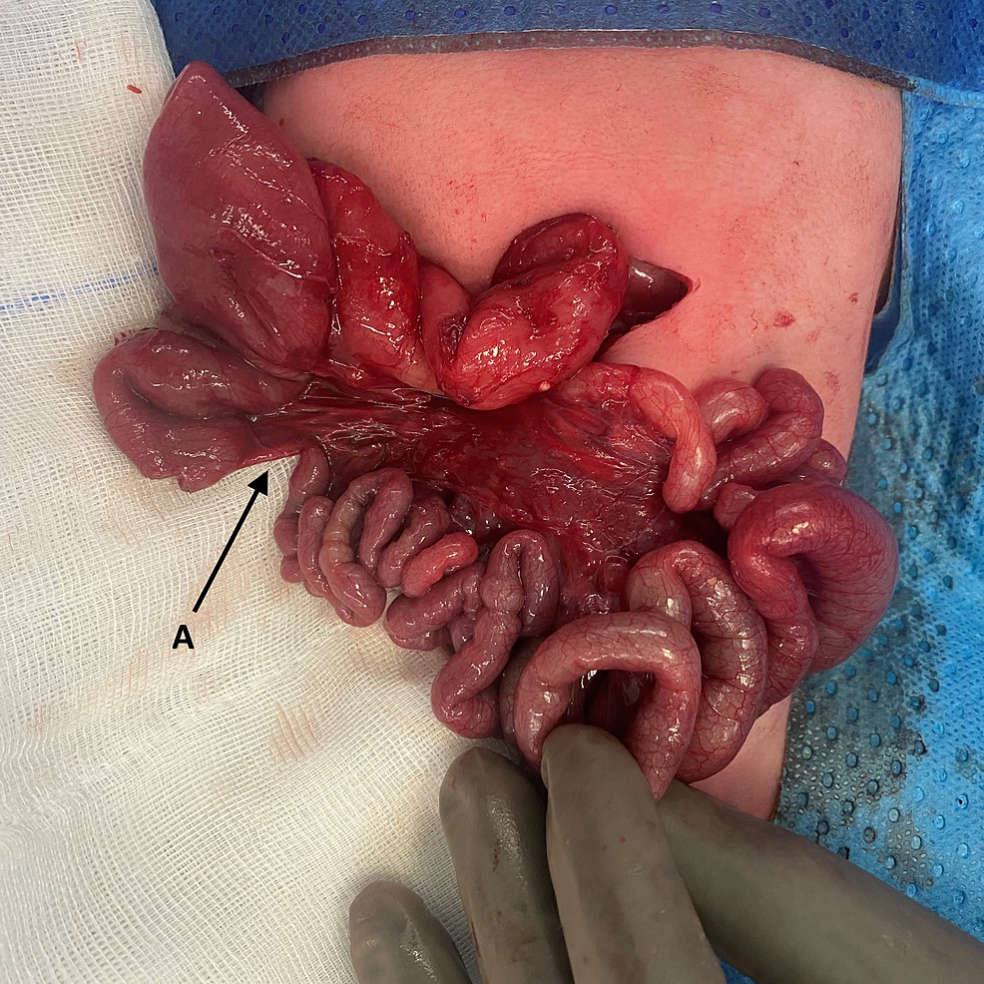
Intraoperative photograph demonstrating the initial finding of a Type II jejunal atresia (A)

**Figure 3:**
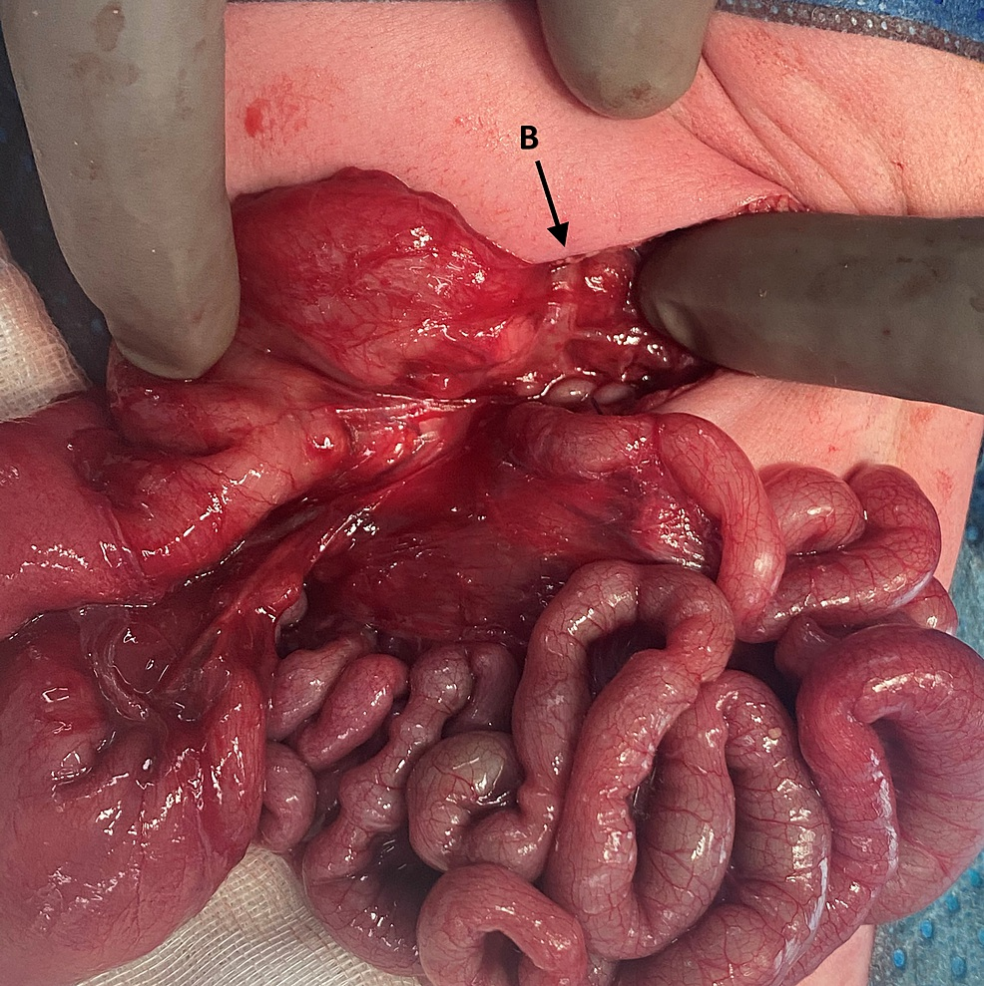
Malrotation and the preduodenal portal vein crossing anterior to the second part of the duodenum (B) were also identified at laparotomy

A laparotomy via a transverse supraumibilical incision was performed. An intestinal malrotation with a narrow mesenteric base, with a PDPV crossing anterior to the second part of the duodenum, and a Type II (Grosfeld classification) jejunal atresia of approximately 5mm in length (Figure 2 and 3) were noted. As there was no suggestion of duodenal obstruction, an intraoperative decision was made to not perform a gastro- or duodenoduodenostomy to correct the PDPV. A Ladd’s procedure with appendicectomy was performed, the atretic jejunal ends were resected and an end-to-end anastomosis performed using 6-0 polydioxanone seromuscular sutures. The eviscerated bowel was returned to the abdomen in a non-rotated orientation.

The patient was extubated on postoperative day 2, and feeding was commenced postoperative day 6. By postoperative day 22, the infant was breastfeeding on demand and able to be discharged home. At 6-month follow-up baby is thriving and had started taking solid foods, with no abdominal concerns.

## Discussion

After the first description in 1921 by Knight, we are aware of 323 cases of PDPV previously described in the literature [1,5]. It is rare for PDPV to be identified as the cause of duodenal obstruction; rather the presence of associated anomalies, such as malrotation, duodenal atresia, and duodenal web are more commonly cause for the obstruction [7]. Esscher reported that while 30% of their PDPV cases were considered to be obstructive, the PDPV was the definitive cause of obstruction in only 5% [3]. This suggests that in the abscess of calibre change at the proximal duodenum, correction of a PDPV may not be required.

A review of the literature demonstrated only one previously reported case of PDPV associated with malrotation and jejunal atresia [6]. In the previously described case, the PDPV was deemed to be causing extrinsic duodenal obstruction, prompting a relook laparotomy, gastroduodenostomy and revision of the initial jejunal anastomosis, in addition to the Ladd's procedure. However, examining the intraoperative photo provided in the manuscript, showing a non-dilated duodenum proximal to the PDPV, we question if the PDPV was the cause of the obstruction. We propose the cause of the obstruction would have been either the non-corrected malrotation, the kinked anastomosis or a combination of the two. Indeed, the authors state in the manuscript that it may be hypothesized that if the initial primary repair of jejunal atresia was successful with permanent relief of duodenal dilatation, the PDPV might have remained asymptomatic.

As there was no clear proximal duodenal obstruction, a corrective procedure for the PDPV was not performed in our case. Gastroduodenostomy and duodenoduodenostomy should not be performed without significant consideration in the neonate as there are potential long-term complications associated such as gastroesophageal reflux, anastomotic stricture and bile reflux gastritis [8,9]. Our decision is supported by similar case reports of intestinal malrotation and incidentally noted PDPV, in which Ladd’s procedure was performed, but the PDPV was left in situ, undisturbed, with no subsequent PDPV-mediated duodenal obstruction [6,10]. The postoperative course for our patient was uneventful which confirms the theory that uncorrected PDPV is asymptomatic and may pose no future threat.

## Conclusions

PDPV rarely causes duodenal obstruction and is usually diagnosed incidentally at the time of surgery for one of its common associations such as malrotation, situs inversus, duodenal stenosis, atresia or web, pancreatic or biliary abnormalities. In the absence of calibre change or evidence of obstruction at the proximal duodenum, PDPV correction is unlikely to be of benefit. We propose that in the rare case of PDPV associated with malrotation and jejunal atresia, surgical correction of the PDPV might not be necessary. 
